# The Cytosolic Phospholipase A_2_α N-Terminal C2 Domain Binds and Oligomerizes on Membranes with Positive Curvature

**DOI:** 10.3390/biom10040647

**Published:** 2020-04-22

**Authors:** Katherine E. Ward, Ranjan Sengupta, James P. Ropa, Souad Amiar, Robert V. Stahelin

**Affiliations:** 1 Department of Chemistry and Biochemistry, University of Notre Dame, South Bend, IN 46556, USA; khoppert@alumni.nd.edu (K.E.W.); jropa@iu.edu (J.P.R.); 2 Department of Medicinal Chemistry and Molecular Pharmacology, Purdue University, West Lafayette, IN 47906, USA; rsengupt@purdue.edu (R.S.); samiar@purdue.edu (S.A.); 3 Purdue Center for Cancer Research, Purdue University, West Lafayette, IN 47907, USA

**Keywords:** cytosolic phospholipase A_2_α (cPLA_2_α), electron microscopy, Golgi, membrane bending, membrane curvature, C2 domain, oligomerization

## Abstract

Group IV phospholipase A_2_α (cPLA_2_α) regulates the production of prostaglandins and leukotrienes via the formation of arachidonic acid from membrane phospholipids. The targeting and membrane binding of cPLA_2_α to the Golgi involves the N-terminal C2 domain, whereas the catalytic domain produces arachidonic acid. Although most studies of cPLA_2_α concern its catalytic activity, it is also linked to homeostatic processes involving the generation of vesicles that traffic material from the Golgi to the plasma membrane. Here we investigated how membrane curvature influences the homeostatic role of cPLA_2_α in vesicular trafficking. The cPLA_2_α C2 domain is known to induce changes in positive membrane curvature, a process which is dependent on cPLA_2_α membrane penetration. We showed that cPLA_2_α undergoes C2 domain-dependent oligomerization on membranes in vitro and in cells. We found that the association of the cPLA_2_α C2 domain with membranes is limited to membranes with positive curvature, and enhanced C2 domain oligomerization was observed on vesicles ~50 nm in diameter. We demonstrated that the cPLA_2_α C2 domain localizes to cholesterol enriched Golgi-derived vesicles independently of cPLA_2_α catalytic activity. Moreover, we demonstrate the C2 domain selectively localizes to lipid droplets whereas the full-length enzyme to a much lesser extent. Our results therefore provide novel insight into the molecular forces that mediate C2 domain-dependent membrane localization in vitro and in cells.

## 1. Introduction

Phospholipases A_2_, which are found in mammals, as well as the venom of snakes, insects, and arachnids, are a large family of enzymes that hydrolyze the bond between the glycerol and second fatty acid chain in phospholipids [1]. Cytosolic phospholipase A_2_α (cPLA_2_α) selectively cleaves phosphatidylcholine containing an *sn*-2 arachidonyl acyl chain, a critical step in the synthesis of prostaglandins and leukotrienes [2]. The analysis of cPLA_2_α knockout mice revealed a marked reduction in arthritis, anaphylaxis, and cerebral ischemia, among other inflammatory diseases, promoting great interest in its catalytic activity and regulation [3,4]. The cPLA_2_α protein has two domains: a ~130-residue N-terminal Conserved 2 (C2) domain and a ~700-residue C-terminal lipase domain [5], the former required for localization to cellular membranes [6]. The C2 domain interacts with lipids in a Ca^2+^-dependent manner [7,8], penetrates 1–1.5 nm into the membrane [9] to enhance membrane resonance time, and selectively binds to the bioactive sphingolipid ceramide-1-phosphate [10,11]. The catalytic domain can be activated by higher cytosolic calcium levels, but C1P plays a key role in cPLA_2_α localization [11,12]. In addition to its role in inflammatory signaling, cPLA_2_α is also important for phagocytosis [13] and Golgi-derived endosomal trafficking [14,15,16]. This homeostatic function is supported by the expression of cPLA_2_α in a wide variety of cell types [3,4].

Several studies suggest a direct role for cPLA_2_α in processes involving membrane curvature, including cholesterol-dependent vesicular transport from the Golgi to the plasma membrane and Golgi intercisternal tubular formations [14,15,16]. Recently, cPLA_2_α was found to be necessary for Fc-mediated phagocytosis in macrophages [13]. Interestingly, this process was independent of cPLA_2_α catalytic activity but dependent on C2 membrane binding [13]. Biophysical studies investigating how proteins generate the membrane curvature necessary for all of these processes are best understood in the context of endocytosis, exocytosis, and vesicular trafficking. The molecular mechanisms responsible for this energetically unfavorable process often involve proteins with peripheral Bin-amphiphysin-Rvs (BAR) or epsin N-terminal homology (ENTH) lipid-binding domains [17]. Although both domains are responsible for inducing and stabilizing membrane curvature, they alter membrane shape via different mechanisms. BAR domains dimerize to form the intrinsically curved structure of a crescent moon, sensing local regions of membrane curvature and forming rigid protein scaffolds to bend and stabilize membrane tubules [18,19]. In contrast, the ENTH domain has a more globular structure and relies on lipid binding to induce conformational changes in an amphipathic α_0_-helix that penetrates the membrane [20,21] and stabilizes the protein–membrane interaction [22]. The ENTH domain was later found to form oligomers on highly-curved membranes via electrostatic residues that are not involved in membrane binding [23]. A computational study found that ENTH domain oligomers on vesicles are less ordered than those found on long tubular structures, providing some insight into the molecular forces that mediate vesiculation and tubulation [24].

Although C2 domains were not initially considered able to induce membrane curvature, several recent studies have provided evidence to support this role. The first C2 domain shown to induce membrane curvature was synaptotagmin-1 [25]. We subsequently showed that cPLA_2_α induces membrane curvature in a calcium-dependent manner, and that both calcium-binding loops must penetrate the membrane to achieve bending [26]. At least four C2 domain proteins have been shown to bend membranes, and three of them contain tandem C2A and C2B domains [25,26,27,28]. Only the C2B domain of synaptotagmin-1 is required to induce membrane curvature (25), whereas only the C2A domain is required in the case of Doc2B, although both C2A and C2B are required for SNARE-dependent vesicle fusion (28). 

To gain insight into the molecular basis of membrane curvature induced by C2 domains, we compared the C2 domain of cPLA_2_α to the well-studied ENTH and BAR domain mechanisms. We found that the C2 domain of cPLA_2_α preferentially localizes to vesicles of high positive curvature (~50 nm in diameter), as previously reported for synaptotagmin-1 and Rasal [25,27]. Additionally, we found that the oligomerization of cPLA_2_α in vitro and in A549 cells is dependent on the C2 domain, and is enhanced on vesicles with greater intrinsic curvature. This report shows that C2 domain oligomerization is used as a mechanism to overcome energetic barriers that prevent the induction and stabilization of curved membranes.

## 2. Materials and Methods 

### 2.1. Materials

The lipids 1-palmitoyl-2-oleoyl-*sn*-glycero-3-phosphocholine (POPC), 1-palmitoyl-2-oleoyl-*sn*- glycero-3-phosphoethanolamine (POPE), 1-palmitoyl-2-oleoyl-*sn*-glycero-3-phosphatidylserine (POPS), 1,2-dioleoyl-*sn*-glycero-3-[(N-(5-amino-1-carboxypentyl)iminodiacetic acid)succinyl] nickel salt (DGS-NTA-Ni), 1-palmitoyl-2-(dipyrrometheneboron difluoride)undecanoyl-*sn*-glycero-3- phosphocholine (TopFluorPC), and 23-(dipyrrometheneboron difluoride)-24-norcholesterol (TopFluorChol) were purchased from Avanti Polar Lipids (Alabaster, AL, USA) and used without further purification. Nunc Lab-Tek II eight-well chambered cover glasses, the Pierce bicinchoninic acid (BCA) protein assay kit and all molecular biology enzymes were sourced from Thermo Fisher Scientific (Waltham, MA USA), with the exception of DNA ligase (New England Biolabs, Ipswich, MA, USA). The mTurquoise2 constructs [29] were a gift from Dorus Gadella (Addgene reference numbers 36204 and 36205).

### 2.2. Protein Purification

The C2 domain sequence of cPLA_2_α in vector pET-28a [7] was expressed and purified as previously described [26]. E116C purification and labeling with Rhodamine Red C2 maleimide (Thermo Fisher Scientific) was carried out according to the manufacturer’s protocol. Briefly, after purification with Ni-NTA resin (Qiagen, Valencia, CA, USA), the protein solution was centrifuged at 50,000× *g* to remove any precipitated protein then gently mixed overnight with a five-fold molar excess of maleimide. The protein was dialyzed three times against 4 L of 20 mM 4-(2-hydroxyethyl)-1-piperazineethanesulfonic acid (HEPES) buffer (pH 7.4) containing 160 mM NaCl to remove free maleimide, and the protein concentration was determined using the Pierce BCA assay. The concentration of maleimide-labeled protein was calculated using a standard curve of seven free maleimide standards prepared using free maleimide and measured on SpectaMax M5 96-well plate reader (λ_excitation_ = 544 nm, λ_emission_ = 590 nm). We achieved 20–40% labeled protein over several purification and labeling protocols. The standard curve for maleimide was used to determine the amount of maleimide in each sample in comparison with the total protein content determined by the BCA assay. Since only one cysteine residue was present in the C2 domain, one label was assumed per C2 domain that was found to be labelled (as a percentage of the total sample).

### 2.3. Cloning and Mutagenesis

**Figure 1:**
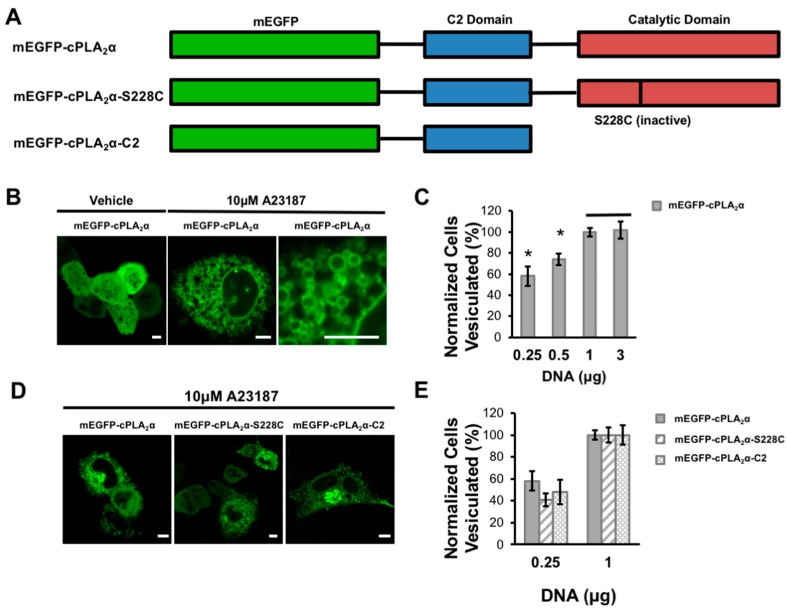
cPLA_2_α induces cellular vesiculation. (**A**) A schematic of the fusion constructs for the monomeric enhanced green fluorescent protein (mEGFP)-cPLA_2_α. (**B**) A549 cells were plated and transfected at 70–90% confluency with varying concentrations of the mEGFP-cPLA_2_α WT or mutant construct. A549 cells were imaged 24 h post transfection and quantified 20–30 min after treatment with either dimethyl sulfoxide (DMSO) vehicle or 10 μM A23187. The vesiculation was defined as cells expressing mEGFP-cPLA_2_α containing >20 cellular vesicles with localized fluorescent protein. The vesiculation was normalized for the total number of cells expressing mEGFP-cPLA_2_α, mEGFP-cPLA_2_α-S228C, or mEGFP-cPLA_2_α-C2. A representative image of mEGFP-cPLA_2_α transfected cells before and after treatment with the calcium ionophore A23187 or DMSO vehicle. (**C**) There were 40–80 cells counted in triplicate to measure the relationship between mEGFP-cPLA_2_α and cellular vesiculation. Data was normalized to the 3 μg DNA average. (**D**) Representative images of mEGFP-cPLA_2_α, mEGFP-cPLA_2_α-S228C, and mEGFP-cPLA_2_α-C2 after treatment with A23187. (**E**) There were 40–80 vesiculated cells quantified (5 replicates performed) and normalized to the 1 μg average. Error bars represent the standard error of the mean and statistics were run using a Student’s t-test. Scale bars = 5 μm; * *p* < 0.04.

The monomeric enhanced green fluorescent protein (mEGFP)-cPLA_2_α sequence in vector pEGFP-C1 was kindly provided by Dr. Charles Chalfant from the University of South Florida [12]. The EGFP-cPLA_2_α (Figure 1A) cassette was removed using BglII and ApaI, and was transferred to vectors pmCherry-C1 and pmEGFP-C1 (Addgene 36412), kindly provided by Dr. Benjamin Glick, University of Chicago, IL, USA. The C2 domain constructs featured a GLRS linker between the mEGFP sequence and the N-terminus of cPLA_2_α (Figure 1A). An EcoR1 site was added to the N-terminus of the C2 domain (residues 1–128) by PCR and the cassette was transferred to vector pmEGFP-C1 using the BglII and EcoR1 sites. Site-directed mutagenesis was carried out using the QuikChange II kit (Agilent Technologies, Santa Clara, CA, USA) according to the manufacturer’s protocol. All constructs and mutations were confirmed by DNA sequencing.

### 2.4. Cell Culture

A549 cells were seeded into Nunc Lab-Tek II eight-well imaging plates at 40–50% confluency in a 50/50 mixture of Dulbecco’s modified Eagle’s medium (DMEM) and Roswell Park Memorial Institute 1640 (RPMI) medium without serum or antibiotics. The cells were cultivated at 37 °C in a 5% CO_2_ atmosphere and were transfected using Lipofectamine-LTX and Plus Reagent (Thermo Fisher Scientific) when they reached 70–90% confluency, according to the manufacturer’s protocol. TopFluor-Cholesterol (TopFluor-Chol) was prepared in a methyl β-cyclodextran (MβCD) delivery system adapted from the protocol established for dehydroergosterol [30]. Briefly, 5 mM TopFluor-Cholesterol was prepared from a chloroform stock solution, dried under nitrogen gas and resuspended in 25 mM MβCD. The mixture was sonicated for 15 min, shaken at 37 °C overnight, then centrifuged at 16,000× *g* for 10 min. The soluble complex was added to A549 cells 21 h after transfection at a concentration of 50 μM and was left in contact with the cells for 3 min before aspiration and replacement with Opti-MEM. 

### 2.5. Confocal Microscopy

The cells were allowed to recover for 3 h after treatment and were imaged by confocal microscopy 24 h post-transfection using a Zeiss LSM 710 confocal microscope fitted with a 63× oil objective with a numerical aperture of 1.4. To quantify the number of cells with vesiculation, cells containing more than 20 cPLA_2_α-localized intracellular vesicles were counted versus the total number of transfected cells (which was determined by counting transfected vs. non-transfected cells in each well). Data were collected in triplicate from independent imaging wells and statistically significant differences were determined using Student’s t-test.

### 2.6. CryoAPEX Method for Localization of cPLA_2_α and Visualization of Morphological Changes of the Golgi Apparatus

HeLa cells were transfected with mEGFP-cPLA_2_α and 15 h post transfection 10 μM of A23187 was added to the media and cells were incubated for 30 more minutes. To observe vesiculation in these cells post treatment, cells were pelleted at 500 × *g* resuspended in 20% BSA and cryofixed using a high pressure freezer (Leica, EM PACT) on copper membrane carriers (Leica). This was followed by freeze substitution (Leica, EM AFS2) in acetone in the presence of uranyl acetate and osmium tetroxide for a period of 4 days. Post freeze-substitution pellet was infiltrated with resin (Durcupan, Sigma-Aldrich) gradually (15%–30%–60%–90%–100%–100% + component C). The membrane carrier containing the pellet was then placed in a mold filled with resin and baked in the oven at 60 °C for 24 h. Copper planchets were then extracted using a razor blade and the blocks were re-embedded with more resin and baked in the oven for another 24 h. Hardened blocks with embedded samples are then trimmed and serially-sectioned using an ultramicrotome. Serial-sections were collected on copper slot grids with formvar support (EM sciences) and imaged using a Tecnai T12 transmission electron microscope operating at 80 kV.

For localization of cPLA_2_α at high resolution, an indirect APEX2 based localization method was carried out. This method was adapted from Ariotti et al. [31]. Briefly, HEK cells were transfected with mEGFP-cPLA_2_α plasmid along with APEX2-csGBP plasmid (A kind gift from Dr. Rob Parton, Addgene #108874) at a 1:1 ratio using Lipofectamine 2000 (Invitrogen) and incubated for 15 h. Cells were treated with 10 μM of A23187 as before and then dislodged from the plates using trypsin and fixed chemically with glutaraldehyde. The pellet was then washed 3 times for 5 min each with 0.1 M sodium cacodylate buffer. The APEX2 peroxidase reaction was then carried out with hydrogen peroxide (in presence of 1 mg/mL 3,3′-diaminobenzamide (DAB) and in sodium cacodylate buffer). Post reaction cells were pelleted and washed pellets were subsequently subjected to the CryoAPEX method as described in Sengupta et al. [32]. Briefly, cells chemically fixed with glutaraldehyde were then cryogenically fixed via high pressure freezing followed by freeze substitution in presence of osmium tetroxide in acetone. Cell pellets were then embedded in resin blocks and baked at 60 °C. Thin (90 nm) serial-sections were then obtained from the resin blocks using a microtome and were collected on formvar coated grids. Grids were loaded onto a Tecnai T12 TEM for image collection.

To visualize the perturbation of the Golgi structure induced by the cPLA_2_α or the C2 domain, HeLa cells constitutively expressing the Golgi cis-medial marker α-mannosidase-II-HRP (kind gift from Dr. Franck Perez, Institut Curie) were transfected with either mEGFP-cPLA_2_α or mEGFP- cPLA_2_α-C2 plasmids and incubated for 15 h. Thirty minutes prior to glutaraldehyde fixation, cells were treated with 10 μM of the calcium ionophore A23187. Cells were detached from the plate using trypsin and centrifuged at low speed to obtain a cell pellet. The pellet was then subjected to the CryoAPEX method [32,33] and processed for EM imaging exactly as above.

### 2.7. Cross-Linking Assay

The POPC stock was dried under a stream of nitrogen and made up to 1.5 mM in 20 mM HEPES (pH 7.4) containing 160 mM KCl. The solution was gently mixed to remove dried lipid from the glass vial. To construct vesicles with different diameters, the following modifications were made to the traditional large unilamellar vesicle (LUV) extrusion protocol: the smallest vesicles were extruded 40 times through a 50-nm filter, the mid-sized vesicles were heated at 37 °C for 10 min and extruded 10 times through a 100-nm filter, and the largest vesicles were heated at 37 °C for 20 min and extruded five times through a 400-nm filter. Vesicle size was confirmed using a Delsa Nano S Particle Analyzer (Beckman Coulter, Brea, CA, USA). The number distribution was used to determine the particle size of each vesicle population in triplicate for each independent experiment. We incubated 35 μL of the 1.5 mM POPC LUV solution with 8 μg cPLA2-C2 in buffer containing either 50 μM CaCl_2_ or 100 μM ethylene glycol-bis(β-aminoethyl)-N,N,N’,N’-tetraacetic acid (EGTA) for 30 min. The protein was crosslinked with 0.5 mM ethylene glycol bis(succinimidylsuccinate) (EGS; Thermo Fisher Scientific) for 5 min, then quenched with 150 mM Tris-HCl (pH 8.0) for 5 min. The samples were separated by 12% SDS-PAGE, stained with Coomassie brilliant blue, destained, and analyzed using ImageJ software.

### 2.8. Giant Unilamellar Vesicle Experiments

Giant unilamellar vesicles (GUVs) were prepared as previously described [26]. The lipids in these experiments were labeled with 0.2% mol TopFluor-PC using the lipid ratio 59.8:20:20:0.2 (POPC:POPE:POPS:TopFluor-PC). The GUVs were incubated in 20 mM HEPES (pH 7.4) containing 160 mM KCl and 50 μM CaCl_2_ to assess their stability. We then added 2 μM cPLA_2_α and incubated the vesicles for 30 min before imaging for confocal microscopy as described above. GUVs containing protein were visualized (λ_excitation_ = 488 nm, λ_emission_ = 561 nm) at similar intensities, as determined using the intensity profile from the Zeiss software. The images were imported into ImageJ to determine the vesicle size, lipid intensity and protein intensity in triplicate for each GUV. The vesicle diameter was measured by drawing a circle around the outer diameter of the GUV and the enclosed area was analyzed for fluorescence intensity in the green and red channels, which together were used to calculate the protein-to-lipid ratio. Each vesicle was measured at three locations to obtain an average protein-to-lipid ratio. The largest GUVs contained budding vesicles, which were highly curved and therefore excluded from the intensity measurements.

### 2.9. Number and Brightness Analysis

The Raster Image Correlation Spectroscopy (RICS) data for Number and Brightness (N&B) analysis was collected on an Olympus FV1000 fitted with a 60× oil PlanApo objective with a numerical aperture of 1.42. The data were collected and exported as previously described [34] and analyzed using SimFCS v2.0. A549 cells were plated and transfected at 70–90% confluency as above, and images were acquired 24 h post-transfection. Cells exposed to ionophore were treated with 10 μM A23187 for 30 min before imaging at a pixel dwell time of 12.5 μs/pixel. The pixel size was set to 50 nm in photon mode as specified in the established protocol. The data were analyzed using simFCS v2.0, where the moving average was subtracted for N&B analysis. The monomer threshold was determined using mEGFP as a control with the same 12.5 μs/pixel dwell time. These N&B settings were then applied to data collected from A549 cells transfected with mEGFP-cPLA_2_α and mEGFP-cPLA_2_α-C2. To quantify the pixel intensities of each average oligomer, colored boxes were added to the data in SimFCS v2.0, where each rectangular box corresponds to the average brightness of the monomer, hexamer, 12-mer, 18-mer, and >18-mer. Each rectangular box was defined with the dimensions 250 × 15 in the cursor window, with the exception of the >18-mer box, which was defined as 150 × 80. The total number of pixels selected under these settings was then compiled from 11 image sets each containing 100 scans. The data were collected from two independent experiments, each independently normalized for the mEGFP control.

### 2.10. Lipid Droplet Staining and Imaging

HeLa cells at a density of 3 × 10^4^ were transfected with EGFP-cPLA_2_α or EGFP-cPLA_2_α-C2 as described above. 15 h post transfection, cells were treated with 5 μM Hoechst and 10 μg/mL Nile Red for DNA and lipid droplet staining, respectively, for 20 min at 37 °C. Media were then removed and cells were treated with 10 μM A23187 or equivalent volume of dimethyl sulfoxide (DMSO) for 30 min at 37 °C. The resulting staining and vesiculation were imaged at 37 °C using a Nikon Eclipse Ti Confocal inverted microscope (Nikon, Japan), using a Plan Apochromat 60× 1.4 numerical aperture oil objective and a 100× 1.45 numerical aperture oil objective, respectively. A 488 nm argon laser line was used to excite EGFP and a 561 nm argon laser was used to excite Nile Red, while a 405 nm argon laser was used for Hoechst excitation. The resulting images were processed and analyzed using ImageJ (http://rsb.info.nih.gov/ij/).

## 3. Results

### 3.1. cPLA_2_α Induces Cellular Vesiculation via its C2 Domain

We investigated the ability of cPLA_2_α to induce vesiculation by transfecting A549 cells with increasing concentrations of mEGFP-cPLA_2_α DNA (Figure 1A). To elevate the cytoplasmic calcium concentration and thus induce cPLA_2_α membrane localization, cells were treated with the calcium ionophore A23187 and imaged by confocal microscopy. As the cellular concentration of cPLA_2_α increased, the number of cells containing more than 20 cytoplasmic vesicles steadily rose until saturation was reached at 1 μg transfected DNA (Figure 1B,C). These data demonstrate the number and generation of cytoplasmic vesicles are dependent upon the expression level of cPLA_2_α.

To determine the role of the cPLA_2_α catalytic and C2 domains in the induction of membrane curvature, we created constructs lacking a functional catalytic domain and tested for vesiculation in transfected A549 cells (Figure 1A). Either deleting the catalytic domain completely (mEGFP-cPLA_2_α -C2) or inactivating it by mutating the catalytic residue Ser^228^ (mEGFP-cPLA_2_α -S228C) caused a slight decrease in vesiculation compared to the wild-type construct in cells transfected with the lowest concentration of cPLA_2_α DNA. These data show that the catalytic domain may contribute to the formation of vesicular structures but that the C2 domain alone clearly has the ability to generate vesicles in cells (Figure 1D,E). At higher concentrations of cPLA_2_α DNA, the constructs lacking a functional catalytic domain were capable of inducing vesiculation to the same degree as the wild-type construct (data not shown).

### 3.2. cPLA_2_α Oligomerizes on Membranes in A549 Cells

**Figure 2:**
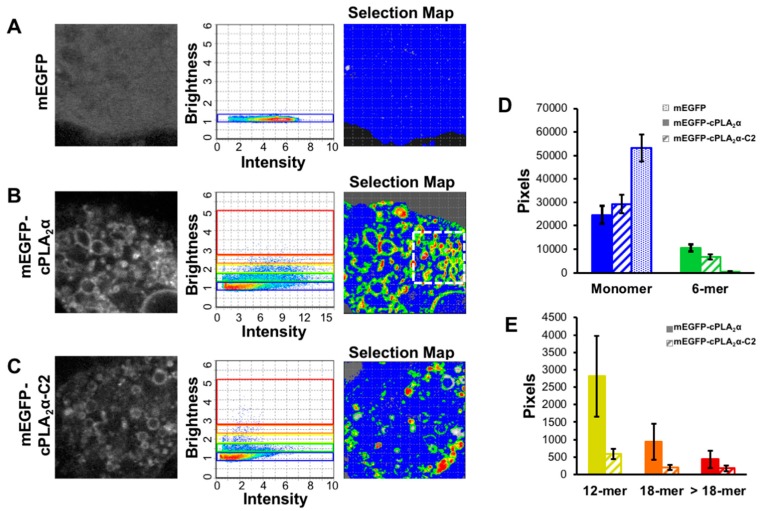
cPLA_2_α oligomerizes on cellular membranes in A549 cells. A549 cells were transfected at 70–90% confluency with mEGFP, mEGFP-cPLA_2_α or mEGFP-cPLA_2_α-C2 for 24 h. All cells were treated with 10 μM A23187 for 30 min then imaged with confocal microscopy. (**A**–**C**) (from left to right) A representative image of Raster Image Correlation Spectroscopy (RICS) data displaying the cytoplasmic localization of fluorescent constructs in A549 cells. Each intensity and brightness plot shows protein oligomerization where increased brightness corresponds to larger protein structures. The selection map displays the selected pixels from the brightness plot mapped back to the cellular morphology. (**D**,**E**) Results were quantified through the analysis of 11 separate cellular data sets from 2 independent experiments using the simFCS software. Graphs display the number of pixels on the *y*-axis and the size of EGFP-tagged protein on the *y*-axis (monomer, 6-mer, 12-mer, 18-mer, and >18-mer). Results are plotted for EGFP (dot filled), EGFP-cPLA_2_α (solid filled), and EGFP-cPLA_2_α-C2 (hash filled).

Next we compared the mechanism of membrane curvature triggered by cPLA_2_α-C2 to that of the ENTH domain. Both domains are known to penetrate membranes [7,8,9,22,35] but it is unlikely that membrane penetration alone can overcome the large energetic barrier that prevents the bending of membranes into highly curved vesicles and their stabilization [17,35,36,37]. The ENTH domain oligomerizes on membranes and selectively binds to vesicles with positive curvature [18,20,23], so we hypothesized that cPLA_2_α also oligomerizes on membranes and tested this hypothesis using N&B analysis based on the RICS protocol in live cells using confocal microscopy [32,38]. A549 cells were transfected with mEGFP, mEGFP-cPLA_2_α, or mEGFP-cPLA_2_α-C2 and imaged 24 h post-transfection. Cells were treated with A23187 to induce calcium influx, which promotes the relocalization of cPLA_2_α to the membrane. The oligomerization data generated using simFCS are summarized as a single image from each dataset (Figure 2A), the intensity versus brightness plot (Figure 2B), and the selection map (Figure 2C). The intensity versus brightness plot reveals that mEGFP generates only monomers in live cells, whereas mEGFP-cPLA_2_α and mEGFP-cPLA_2_α-C2 produce many bright pixels corresponding to large protein complexes. These larger pixels were then mapped back to the cell in the selection map, which displays the higher-order structures localized at highly curved regions inside the cell. The white dashed box in the selection map (Figure 2B) clearly shows that cPLA_2_α localizes and oligomerizes on highly-curved vesicles rather than larger vesicles in the same cell. Although more pronounced for the mEGFP-cPLA_2_α construct, the mEGFP-cPLA_2_α-C2 construct was also able to generate oligomers in the absence of the catalytic domain.

To quantitate these results, 11 separate datasets were collected, normalized, and analyzed for oligomerization (Figure 2D,E). We found that mEGFP-cPLA_2_α consistently generated more protein oligomers than mEGFP-cPLA_2_α-C2. The deletion of the catalytic domain reduced the enzyme’s ability to form a vast number of vesicles, and may have contributed to the slight reduction in cPLA_2_α oligomerization, further supporting a potential role for the catalytic domain in this process. The quantitative data also provide evidence that the C2 domain plays an important role in the formation of oligomers, given the presence of oligomers in the cells transfected with mEGFP-cPLA_2_α-C2, which completely lack the catalytic domain. Based on the N&B data, we propose that the C2 domain is likely to drive oligomerization in these cells.

### 3.3. cPLA_2_α-C2 Oligomerizes on Lipid Vesicles

**Figure 3:**
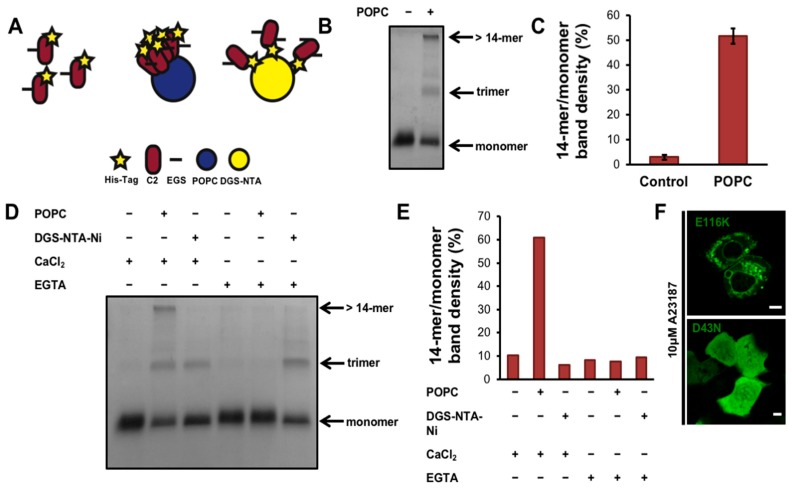
The cPLA_2_α C2 domain oligomerizes on lipid vesicles. (**A**) schematic of the crosslinking assay displaying the possible experimental outcomes. First, C2 domain with label is highlighted in the left panel of A, followed by the C2 domain binding to vesicles (POPC in blue), which would have captured oligomers in the presence of crosslinker (black line). Alternatively, at less protein density on the membrane surface, as in the presence of 1,2-dioleoyl-*sn*-glycero-3-[(N-(5-amino-1-carboxypentyl)iminodiacetic acid)succinyl] nickel salt (DGS-NTA) (right panel of A), C2 oligomers are proposed to be in lower quantity. (**B**) 35 μL of 1.5 mM 1-palmitoyl-2-oleoyl-*sn*-glycero-3-phosphocholine (POPC) large unilamellar vesicles (LUVs) were incubated with 8 μg of cPLA_2_α-C2 in 20 mM 4-(2-hydroxyethyl)-1-piperazineethanesulfonic acid (HEPES) (pH 7.4), 160 mM KCl and 50 μM CaCl_2_ for 30 min. The protein was crosslinked using 0.5 mM ethylene glycol bis(succinimidylsuccinate) (EGS) for 5 min, then the reaction was quenched with 150 mM Tris (pH, 8.0) for 5 min. The samples were run on 12% SDS-PAGE, stained with Coomassie blue, destained for 120 min, and analyzed with ImageJ. (**C**) Quantification of crosslinking experiments was completed in triplicate and normalized by dividing the >14-mer band density to the monomer band density as determined with ImageJ. (**D**) POPC or 1,2-dioleoyl-*sn*-glycero-3-[(N-(5-amino-1-carboxypentyl)iminodiacetic acid)succinyl] nickel salt (DGS-NTA-Ni) lipids were incubated with buffer either containing 50 μM CaCl_2_ or 100 μM ethylene glycol-bis(β-aminoethyl)-N,N,N’,N’-tetraacetic acid (EGTA) to chelate calcium. Each mixture was resolved using SDS-PAGE, stained with Coomassie blue and destained. (**E**) A representative quantification of D, which was performed independently in duplicate. Data was normalized as stated in C. (**F**) A549 cells were transfected at 70–90% confluency with either mEGFP-cPLA_2_α-E116K or mEGFP-cPLA_2_α-D43N and imaged 24 h post transfection. Cells were treated with 10 μM A23187 for 30 min and representative images are shown.

We investigated the role of the cPLA_2_α-C2 domain in more detail using the molecular cross-linker EGS to covalently trap protein oligomers for analysis by SDS PAGE [39]. To determine whether oligomers could be detected, purified cPLA_2_α-C2 in the presence of calcium with or without POPC liposomes was allowed to react with the crosslinker EGS for 5 min (Figure 3B,C). We found that cPLA_2_α-C2 forms both trimers and larger protein complexes (with more than 14 subunits). To control for random diffusion within membranes and the restriction of the C2 domain to two dimensions, we designed an experiment using DGS-NTA-Ni lipids, which recruit His_6_-tagged proteins to membranes. Liposome-bound proteins are restricted to two dimensions, in contrast to proteins in solution, which can move in three dimensions. The cPLA*_2_*α-C2 His_6_ tag was used to recruit the protein to membranes in a nonspecific manner (Figure 3A,D,E). These results clearly showed that, in the presence of calcium and POPC liposomes, EGS traps cPLA*_2_*α-C2 in an oligomer with at least 14 subunits. In comparison, the DGS-NTA-Ni control experiments generated a nonspecific trimer band, which can be attributed to an artifact of the protein diffusing within the membrane during the short incubation with EGS. Quenching the calcium with EGTA abolished the POPC-dependent 14-mer but not the nonspecific trimer, confirming these results. Taken together, these data support the cellular N&B experiment showing that cPLA_2_α oligomerizes on membranes via its C2 domain in a calcium-dependent manner.

### 3.4. cPLA_2_ Selectively Binds to and Oligomerizes on Membranes with Increasing Positive Curvature

**Figure 4:**
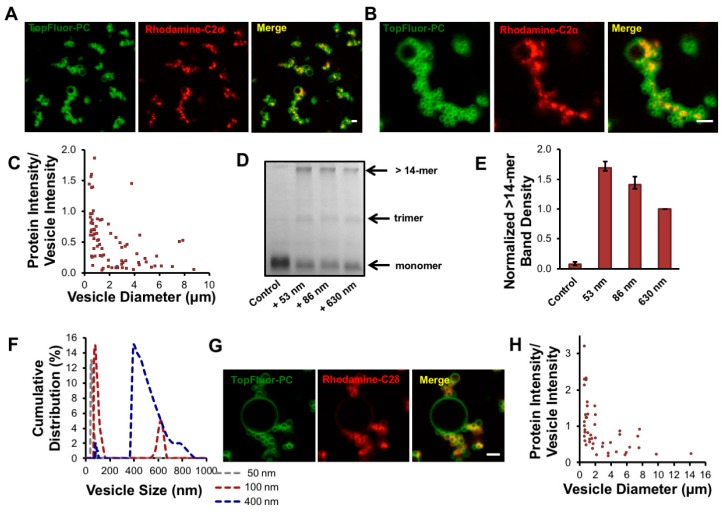
cPLA_2_α preferentially binds to highly curved membranes. (**A**,**B**) TopFluorPC labeled giant unilamellar vesicles (GUVs) with the lipid ratio (59.8:20:20:0.2) (POPC:POPE:POPS:TopFluorPC) were incubated with 2 μM cPLA_2_α-C2-E116C labeled with Rhodamine Red^®^ C_2_ Maleimide (Rhodamine-C2α) for 30 min then imaged with confocal microscopy. (**C**) Protein intensity bound to lipids was normalized to the vesicle intensity in triplicate for vesicles ranging from 500 nm to 15 μm in size as specified in the methods section. Vesicle size and intensity values were measured using ImageJ. (**D**) The crosslinking experiment was repeated as stated in Figure 3B with POPC vesicles in buffer containing 20 mM HEPES, 160 mM KCl, 50 μM CaCl_2_, pH 7.4. (**E**) Band density of the band >14-mer was quantified from D using ImageJ and normalized to the >14-mer band density for 630 nm vesicles. (**F**) The number distribution of the vesicles used in D and E as measured with dynamic light scattering. (**G**) 2 μM cPLA_2_δ-C2 labeled with Rhodamine Red^®^ C_2_ Maleimide (Rhodamine-C2δ) was incubated with GUVs as stated in A. (**H**) Quantification was completed as stated in C. Scale bars are 2 μm and error bars represent the standard error of the mean.

To determine the mechanism of membrane bending by cPLA_2_α, we investigated the relationship between membrane binding and oligomerization in vitro. To visualize cPLA_2_α-C2 by confocal microscopy, we introduced a point mutation at E116 to provide a thiol group for labeling with the reactive fluorophore Rhodamine Red C_2_ maleimide. The E116C mutation had no effect on the localization of cPLA_2_α or its ability to form intracellular vesicles (Figure 3F). To visualize membrane curvature induced by cPLA_2_α-C2, we incubated POPC:POPE:POPS:TopFluorPC GUVs with cPLA_2_α-C2-E116C-Rhodamine (Rhodamine-C2). The labeled C2 domain clearly induced membrane curvature, but selectively localized to vesicles with a small diameter (Figure 4A,B). To quantify this effect, we measured the protein and lipid content of more than 80 vesicles, each based on more than 20 images (Figure 4C). The results showed that Rhodamine-C2 preferentially localized to GUVs less than 2 μm in diameter.

Given the refractive index limitations of confocal microscopy, we were unable to collect data for GUVs smaller than 500 nm in diameter. We hypothesized that cPLA_2_α not only binds more efficiently to highly-curved vesicles, but also undergoes more effective oligomerization in this setting. To determine whether the curvature-sensing trend continued for liposomes smaller than 500 nm in diameter, and to confirm our observation that oligomerization was enhanced on vesicles in A549 cells, cPLA_2_α-C2 oligomerization was measured using the EGS cross-linking assay with vesicles averaging 53, 86, and 630 nm in diameter. The average vesicle size was measured by dynamic light scattering. In addition, using the apparent *K*_d_ and the surface area of the cPLA_2_α protein and liposomes, we calculated an occupied surface area far lower than the 20% coverage needed to induce curvature by membrane crowding [37]. These calculations revealed less than 7% surface area variation among the various vesicle populations, which cannot explain the large fold-change observed in this assay. We observed a significant difference (*p* < 0.05) in the degree of cPLA_2_α-C2 oligomerization between 630-nm vesicles and both the 53-nm and 86-nm vesicles (Figure 4D,E,F). A reproducible difference in oligomerization was also observed between the 53-nm and 86-nm vesicles, although the difference was not statistically significant (*p* = 0.052). To determine if C2 domains from other cPLA_2_ isoforms can induce and/or sense membrane curvature, we expressed, purified and labelled the C2 domain of cPLA_2_δ, which selectively localized to regions of positive curvature as shown in Figure 4G,H.

### 3.5. cPLA_2_α Induces Vesiculation from the Golgi in Cholesterol-Rich Vesicles

**Figure 5:**
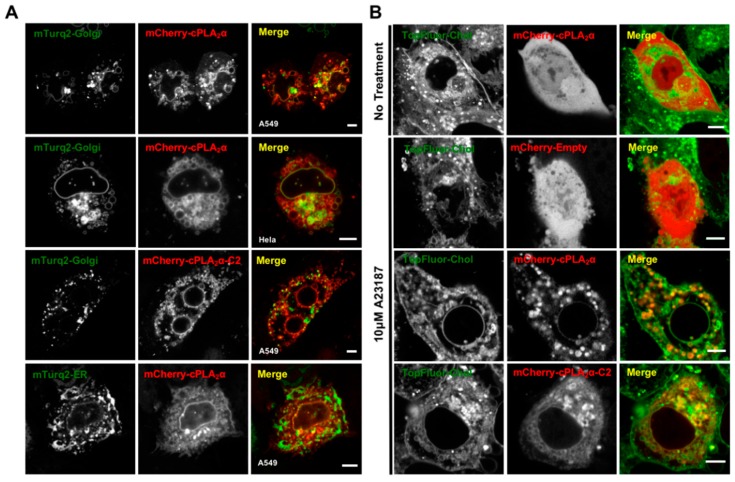
cPLA_2_α vesiculates Golgi membranes and induces vesiculation in cholesterol enriched membranes. (**A**) A549 or HeLa cells were transfected with either mTurquiose2-Golgi or mTurquoise2-ER and mCherry-cPLA_2_α or mCherry-cPLA_2_α-C2 for 24 h, treated with 10 μM A23187 for 30 min and imaged with confocal microscopy to analyze cellular localization. (**B**) A549 cells were transfected with mCherry-Empty (mCherry-lacking a fusion protein), mCherry-cPLA_2_α or mCherry-cPLA_2_α-C2 for 21 h, then treated with 50 μM TopFluorChol (from the MβCD complex) for 3 min. Fresh medium was applied and the cells were incubated at 37 °C for 3 h. Cells were then treated with 10 μM A23187 for 30 min and imaged for vesicle localization. All scale bars are 5 μm and the images were selected as representative images of the data collected.

The cPLA_2_α protein consistently localizes to the highly-dynamic structure of the Golgi tubular and vesicular network [6,15,39], which is not thought to be PI(4)P-dependent like some Golgi targeted proteins [40]. Thus, we investigated the morphology of the Golgi body in A549 and HeLa cells transfected with the cPLA_2_α construct. As markers, we used the Golgi-localized fusion construct mTurquoise2-Golgi [29] (Figure 5). We found that the mCherry-cPLA_2_α fusion protein localized to the Golgi (Figure 5) and induced vesiculation in both A549 and HeLa cells (Figure 5A). The same activity was observed for the construct mCherry-cPLA_2_α-C2, showing that the C2 domain alone is capable of both localizing to the Golgi and inducing vesiculation (Figure 5B). To control for the overexpression of cPLA_2_α and potential nonspecific ER localization and membrane bending, we expressed the marker mTurquoise2-ER [29] but observed little co-localization in A549 cells (Figure 5A). These results, together with previous reports describing cPLA_2_α membrane trafficking [14,15], support our hypothesis that cPLA_2_α-dependent vesiculation originates from the Golgi.

An alternative cholesterol-dependent mechanism for cPLA_2_α-dependent membrane bending has been proposed based on a series of elegant cell-based experiments [14,16]. To determine whether these results translate into our experimental system in A549 cells, we treated the cells with TopFluor-Cholesterol (TopFluor-Chol), a fluorescent derivative of cholesterol that has similar phase transition properties to native cholesterol and is trafficked in a similar manner [41]. We found that cells treated with calcium ionophore generated cytoplasmic vesicles containing TopFluor-Chol co-localized with mCherry-cPLA_2_α or mCherry-cPLA_2_α-C2 (Figure 5B). These data support our hypothesis that cPLA_2_α-induced vesiculation occurs via a cholesterol-dependent mechanism in A549 cells and may be induced by the cPLA_2_α C2 domain.

### 3.6. CryoAPEX Method for Transmission Electron Microscopy Visualization of cPLA_2_α Localization

**Figure 6:**
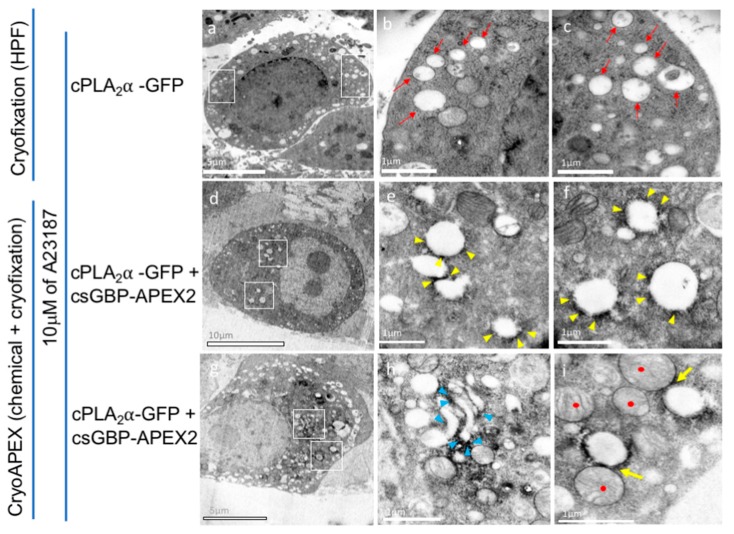
Cells ectopically expressing cPLA_2_α in the presence of calcium ionophore exhibit extensive vesicular structures with detectable presence of cPLA_2_α on vesicles and Golgi. (**a**) Cryogenically fixed (HPF) HeLa cells expressing cPLA_2_α when exposed to 10 μM A23187 results in the accumulation of vesicular structures in the cytoplasm. At higher magnification of the areas (**a**, white boxes) these vesicles show lack of density within and exhibit size heterogeneity (red arrows, **b** and **c**). (**d**) Indirect cryoAPEX using an anti-GFP nanobody (APEX2-csGBP) tagged with APEX2 protein detected density corresponding to cPLA_2_α-GFP in the cytoplasm at lower magnification. (**e**) At higher magnifications the APEX2 specific osmium staining was apparent around these vesicles (**e** and **f**, yellow arrowheads) indicating the concentration of cPLA_2_α in consonance with confocal imaging presented earlier. Stacked membrane bound structure resembling that of Golgi complex were also stained with apex2 specific osmium, (**g** and **h**, blue arrowhead). These stained vesicles were often found to be closely associated with mitochondria (i, yellow arrow; red dots = mitochondria).

**Figure 7:**
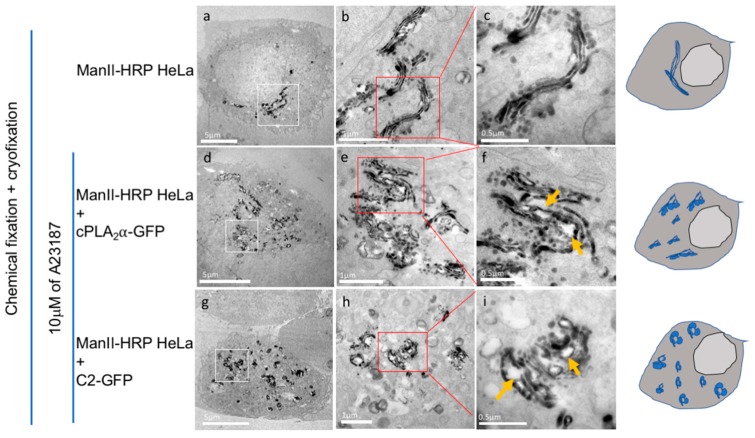
Ectopic cPLA_2_α and/or C2 domain expression in the presence of A23187 induces vacuolization, fragmentation, and dispersion of the Golgi apparatus. (**a**–**c**) A HeLa stable cell line expressing mannosidase-II-HRP was used to image changes in Golgi apparatus morphology. A hybrid chemical-cryo fixation method was employed to demonstrate a typical perinuclear Golgi appearance and high quality of preservation of Golgi architecture in untreated control cells (**b** is a magnified image of **a** and **c** a magnification of the Golgi stack within red box in (**b**). (**d**–**f**) Ectopic expression of cPLA_2_α in cells expressing the Golgi marker shows dispersion of Golgi from its typical perinuclear location. At higher magnification (**e** and **f**), these dispersed Golgi appear to be mini-stacks with extensive herniation and vacuolization of the cisternae (**e**, orange arrows). (**g**–**i**) In cells ectopically expressing isolated cPLA_2_α C2 domain, a clear dispersion of Golgi is visualized. At higher magnification, these dispersed Golgi appear to have almost completely lost cisternal structure with wide spread vesiculation of the ministacks (**h** and **i**, orange arrows). Cartoons of cells at the end of each row of image are provided to help summarize these results.

In order to gain more detailed insight into cPLA_2_α localization and vesicle formation, we imaged HeLa cells expressing mEGFP-cPLA_2_α co-expressed with a conditionally stable APEX2-GFP (csGBP-APEX2) binding nanobody [42] (Figure 6). The conditionally stable APEX2 allows for identification of protein localization following the conversion of the DAB substrate to precipitation of a DAB polymer, which is locally deposited [43]. HeLa cells expressing mEGFP-cPLA_2_α and treated with calcium ionophore (Figure 6a), exhibited extensive vesicular structures that at high magnification (Figure 6b,c) demonstrate size heterogeneity and a lack of density within the vesicular structures. The indirect cryoAPEX was next applied using the aforementioned anti-GFP nanobody (csGBP-APEX2), which detected the APEX2 specific staining density corresponding to mEGFP-cPLA_2_α around these vesicular structures (Figure 6d–i). Further, these stained vesicles were often found to be closely associated with mitochondria (Figure 6g–i, yellow arrow; red dots = mitochondria). A membrane bound structure resembling that of the Golgi complex was also stained with APEX2 specific osmium, (Figure 7h, blue arrowhead), in agreement with cPLA_2_α localization to the Golgi complex previously presented herein and in previous studies [14,15,44].

### 3.7. CryoAPEX Method for Transmission Electron Microscopy Visualization of cPLA_2_α Localization

The cryoAPEX method was also used to investigate changes to the Golgi apparatus following an increase in cytosolic calcium levels in cells ectopically expressing mEGFP-cPLA_2_α or the mEGFP-cPLA_2_α-C2 protein constructs (Figure 7). For these purposes a HeLa stable cell line constitutively expressing the Golgi cis-medial marker α-mannosidase-II-HRP [45] was used to probe changes in appearances of the Golgi. Expression of mEGFP-cPLA_2_α and an increase in the cytoplasmic calcium levels led to extensive dispersion of the Golgi from its typical perinuclear location (Figure 7a–f). At higher magnification, the dispersed Golgi appears to be mini-stacks with extensive herniation and vacuolization of the cisternae (Figure 7e,f, orange arrows). Similarly, in cells ectopically expressing the isolated cPLA_2_α C2 domain, a clear dispersion of the Golgi was detectable (Figure 7g). At higher magnification, these dispersed Golgi appear to have almost completely lost cisternal structure with wide spread vesiculation of the ministacks (Figure 7h,i, orange arrows). In agreement with cell imaging presented above, either the C2 domain or full-length cPLA_2_α were sufficient to disrupt host cell Golgi architecture and increase vesicular formation in cells, where vesicles were enriched with the presence of cPLA_2_α.

### 3.8. Lipid Droplet Imaging for cPLA_2_α and cPLA_2_α-C2 Localization

**Figure 8:**
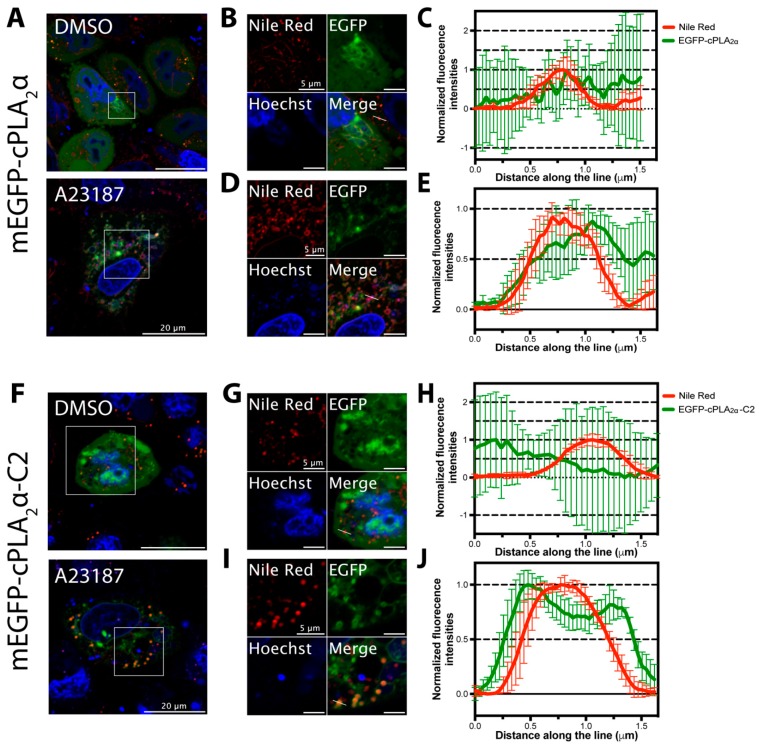
The cPLA_2_α-C2 domain was enriched on lipid droplet membranes following an increase in cytosolic Ca^2+^. HeLa cells were transfected with either EGFP-cPLA_2_α (**A**,**B**,**D**) or EGFP-cPLA_2_α-C2 (**F**,**G**,**I**) for 15 h, treated with 5 μM Hoechst (DNA staining) and 10 μg/mL Nile Red (lipid droplets staining) for 20 min, and treated with 10 μM A23187 or equivalent volume of DMSO for 30 min at 37 °C then imaged with confocal microscopy. **B** and **D**) are zoomed insets of EGFP-cPLA_2_α in **A**, cells treated with DMSO and A23187, respectively. **C** and **E**) are representative plot profiles of fluorescence intensity distributions of both Nile red and EGFP along the white line indicated in **B** and **D**, respectively. **G** and **I** are zoomed insets of EGFP-cPLA_2_α-C2 in **F**, treated with DMSO and A23187, respectively. **H** and **J**) are representative plot profiles of fluorescence intensity distributions of both Nile red and EGFP along the white line indicated in **G** and **I**, respectively. Scale bars: 20 µm in **A** and **F**, 5 µm in **B**, **D**, **G**, and **I**.

cPLA_2_α has been shown to localize to lipid droplets [46,47] and also be critical to lipid droplet synthesis in monocytes [48]. To test whether cPLA_2_α or the cPLA_2_α-C2 domain were able to translocate to lipid droplet membranes upon Ca^2+^-dependent protein translocation, we assessed protein localization using the lipid droplet stain Nile Red upon treatment with the ionophore A23187 or DMSO. As shown in Figure 8, DMSO-treated cells exhibited a diffuse cytoplasmic distribution of the EGFP tagged protein (Figure 8A,F) with some tubular perinuclear structures as described previously for cPLA_2_α [15] or some protein accumulation in the nucleus and cytosol for the cPLA_2_α-C2 domain, however, in both cases no colocalization with lipid droplet stain (Nile Red) was observed (Figure 8B,G, respectively). Plot profile of fluorescence intensities of the EGFP and Nile Red signals along the white line across the lipid droplets showed a uniform distribution of the EGFP fluorescence while Nile Red exhibited higher intensity on the center of the line that corresponded to the center of lipid droplets (Figure 8C,H). Upon the induction of vesiculation and protein translocation with the ionophore A23187, EGFP-cPLA_2_α showed a low level of localization to lipid droplet membranes (Figure 8A lower panel, and 8D) and the plot profile of both fluorescence intensities indicated that the protein may bind nonuniformly to lipid droplet membranes (Figure 8E). However, the catalytically inactive EGFP-cPLA_2_α-C2 protein exhibited a clear Ca^2+^-dependent translocation to lipid droplet membranes (Figure 8F lower panel, and I). Furthermore, the plot profile of EGFP and Nile red fluorescence intensities indicated a uniform distribution of C2 domain fluorescence across lipid droplet membranes. The enhanced C2 domain localization to lipid droplets but not that of cPLA_2_α may best correlate with the C2 domain selectivity of membranes with positive curvature and oligomerization on those membranes.

## 4. Discussion

We investigated the role of the C2 domain in the membrane curvature induced by cPLA_2_α in order to gain insight into the underlying molecular mechanisms. We found that cells transfected with mEGFP-cPLA_2_α showed pronounced vesiculation after treatment with the calcium ionophore A23187 (Figure 1). Although cPLA_2_α is known to form tubules, and this process is dependent on its catalytic activity [16], cPLA_2_α has also been observed to promote vesiculation in HeLa cells [14]. However, we are the first to show that this process can occur in live cells expressing cPLA_2_α lacking a functional catalytic domain (Figure 1). We observed some tubulation (data not shown), but cells containing a vast number of cytosolic vesicles were the predominant phenotype (Figure 1, Figure 5 and Figure 7). To explore the molecular forces contributing to C2 domain-dependent membrane curvature in more detail, we used N&B analysis to measure oligomerization in real time. We observed cPLA_2_α oligomerization in A549 cells, but also found that the cPLA_2_α-C2 construct lacking the catalytic domain was also able to induce vesiculation and undergo oligomerization in cells, albeit to a less degree than the wild-type construct (Figure 2). By analyzing multiple datasets, we found that localization and oligomerization were concentrated at cellular regions featuring a high degree of curvature.

We therefore hypothesized that cPLA_2_α can sense curvature and preferentially undergo oligomerization on highly-curved vesicles. Our in vitro experiments confirmed these results, given that cPLA_2_α-C2 preferentially localized and induced curvature on vesicles ~50 nm in diameter, rather than those with diameters of ~80 or ~600 nm. To determine the physiological relevance of these findings, we investigated cPLA_2_α-induced membrane bending in the context of Golgi localization and cholesterol trafficking, finding that cPLA_2_α and cPLA_2_α-C2 localized to Golgi-derived vesicular membranes that were rich in cholesterol. These results are consistent with earlier findings [14,16], confirming the physiological relevance of membrane curvature induced by the C2-domain. Golgi-derived COPI and COPII vesicles are similar in size and curvature to the preferential targets for cPLA_2_α-C2 membrane curvature and oligomerization [46]. Considering other reports suggesting the role that the catalytic domain plays in the generation of tubulation in cells, it is tempting to hypothesize that the C2 and catalytic domains work together to maintain a balance between vesicles and tubules to define the structure of the Golgi membrane.

Compared to other lipid-binding domains that can bend membranes, cPLA_2_α-C2 is unique because it contains a C2 domain that penetrates membranes to a depth of 1–1.5 nm [9] in addition to a catalytic domain that generates lysophospholipids. The α_0_-helix of the epsin ENTH domain penetrates the membrane to a depth of 0.2–0.5 nm [35] whereas most of the C2 domain lipid-binding surface penetrates into the membrane. We previously confirmed that membrane bending by cPLA_2_α-C2 was dependent on deep membrane penetration using a series of hydrophobic mutants lacking the ability to penetrate or bend the membrane in several model lipid systems [26]. Unlike other lipid-binding domains that induce membrane curvature, the cPLA_2_α catalytic domain converts phosphatidylcholine with an *sn*-2 arachidonyl chain to lysophosphatidylcholine and arachidonic acid. The production of lysophosphatidylcholine and release of arachidonic acid generates cone-shaped lipids, which are known to induce positive membrane curvature [49]. Our cell-based assays confirmed the importance of catalytic activity to membrane bending (Figure 1). In addition, we found evidence that supports the hypothesis that the C2 domain senses curvature and oligomerizes on the membrane to contribute to the generation of membrane curvature (Figure 2, Figure 3 and Figure 4). 

**Figure 9:**
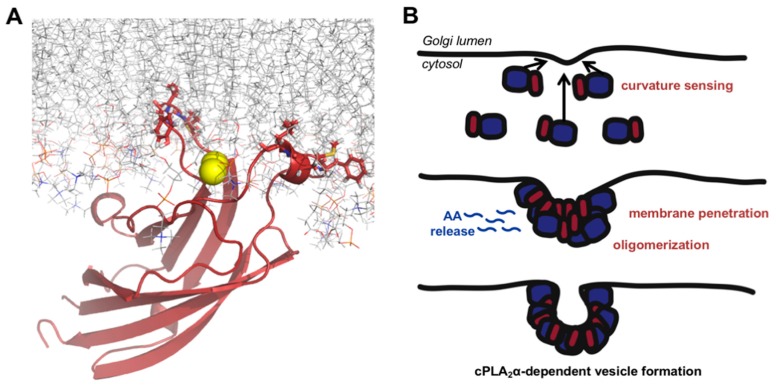
Mechanistic hypothesis of cPLA_2_α induced membrane curvature changes, curvature sensing and vesicle formation. (**A**) cPLA_2_α-C2 (PDB 1CJY) shown penetrating into a POPC bilayer. The structure of the C2 domain is displayed as a ribbon in red, the hydrophobic penetrating residues are shown as red sticks and the POPC molecules are shown as lines. The sticks and lines are colored so that the hydrogen atoms are light grey, nitrogen atoms are blue, and oxygen atoms are light red. (**B**) A schematic of the current model for the generation of cPLA_2_α-induced membrane curvature with contributions from membrane penetration, protein oligomerization and lipid metabolism of acyl chains at the sn-2 positions to produce arachidonic acid (AA) and lysophospholipid. Panel A was generated with Pymol while panel B was drawn using Adobe Illustrator.

Our results indicate the following mechanism for cPLA_2_α-induced membrane curvature (Figure 9). There is a 70–300 kcal/mol energetic barrier that must be overcome to induce and stabilize substantial curvature [17]. Therefore, we propose that cPLA_2_α senses local regions of curvature and preferentially binds to these curved regions when calcium influx occurs within the cytoplasm. Targeting likely requires C1P in the Golgi, because basic residues in the β-groove greatly reduce localization [11,12]. It is therefore plausible that these regions are C1P-enriched in A549 and Hela cells. The binding of cPLA_2_α not only stabilizes the newly-curved membrane, but also allows the C2 domain to penetrate deeply into the hydrocarbon core of the bilayer to destabilize and bend the membrane. The stronger affinity for highly-curved membranes recruits additional cPLA_2_α to the membrane, allowing the C2 domain to form oligomers and induce further membrane curvature. In addition to the actions of the C2 domain, the moment cPLA_2_α arrives at the membrane the catalytic domain begins to generate the conical lysophosphatidylcholine and releases arachidonic acid from the membrane, promoting positive curvature. To our knowledge, this is the first curvature-inducing mechanism of its kind, with potential cooperation between a lipid-metabolizing domain and a lipid-binding domain. However, cPLA_2_α has recently been shown via sophisticated lipidomics and molecular dynamics analysis to exist in a bound and unbound form at the membrane [50] and our current results cannot decipher the difference in these two conformations on membrane curvature generation.

Membrane curvature-inducing domains generate and stabilize curvature via complex mechanisms. The epsin ENTH domain was shown to bind PI(4,5)P_2_ in the plasma membrane [20], causing a conformational shift that allows it to penetrate the membrane with its amphipathic α-helix [20]. The importance of membrane penetration is difficult to isolate from the experimental findings because mutating the membrane-penetrating hydrophobic and aliphatic amino acids also reduces the lipid-binding affinity of the domain [22,26]. Two recent studies used a model system with the synthetic lipid analog DGS-NTA-Ni [36,37], which interacts with His_6_-tagged proteins independently of membrane penetration. Both reports cast doubt upon the importance of membrane penetration by the epsin ENTH domain in the generation of membrane curvature. Although this is an incredibly powerful system to recruit proteins to lipid membranes, the experiments assume the biophysical properties of this long, bulky, charged head group behave like native lipid head groups. Considering the biophysical evidence showing that conical lipids are susceptible to the generation of spontaneous positive curvature [49] and the fact that the structural properties of DGS-NTA-Ni have not been compared to more biologically-relevant lipids, these findings should be verified in a more biologically-relevant system.

We provided the first evidence for C2 domain-dependent membrane bending based on a penetration-dependent mechanism [26]. We also showed that membrane penetration by calcium-binding loops 1 and 3 (previously shown to penetrate 1–1.5 nm into the membrane) promote the generation of membrane curvature, and both loops were essential for this process. Others have shown that the calcium-binding loops of synaptotagmin-1 C2A and C2B domains penetrate the membrane, yet only the C2B domain induces curvature [24]. Recently, Doc2B was also shown to generate membrane curvature, but only the C2A domain is strictly necessary [26]. The difference in structure–function relationship between these proteins suggests that the C2 domains must be able to adopt different confirmations that are capable or incapable of inducing membrane curvature. It is tempting to hypothesize that these C2 domains may also oligomerize on the membrane to stabilize membrane curvature. Oligomerization may help these C2 domains overcome the energetic barrier to form vesicles and tubules, while the tandem C2 domain may serve other purposes such as the regulation of membrane bending via protein–lipid or protein–protein interactions that contribute to the protein’s overall function in cells. In support of this hypothesis, the recent structure elucidation of the cPLA_2_α C2 domain bound to phosphatidylcholine and calcium contained three C2-domains in the asymmetric crystal unit [51]. Moreover, two asymmetric crystal units contained a total of six C2 domains, which demonstrated PC-binding increased C2 domain packing to form a tube-like structure. The authors of this seminal study suggest this crystal structure packing supports the notion of C2-induced positive membrane curvature changes (Figure 9), which couldn’t previously be well visualized by crystal structures available for the non-lipid bound cPLA_2_α C2 domain [8,52]. This recent crystal structure of the lipid bound C2 domain also supports the lipid-induced oligomerization [51] we have detected in vitro and in cells with the C2 domain and cPLA_2_α.

The membrane penetration residues of the epsin ENTH domain were recently shown to play another role in the generation, stabilization, and fission of vesicles. In contrast to the BAR domain, which stabilizes membrane curvature by generating rigid protein scaffolds [18,53], membrane penetration by the ENTH domain induces vesicle fission by destabilizing the vesicle neck [18]. This effect was enhanced by increasing the size of the membrane-penetrating hydrophobic residue [18]. Interestingly, enhancing the hydrophobicity of the membrane-penetrating loops in a simple system containing only synaptotagmin-1 causes the protein to generate vesicles less than 10–20 nm in diameter, suggesting that, like the epsin ENTH domain, membrane penetration by C2 domains may also promote vesicle fission independently, and future experiments should explore this possibility [24]. 

The cPLA_2_α protein was shown to localize to the Golgi, primarily the trans-Golgi network [15,44], which occurs in response to calcium influx and selective binding to C1P [10,11,12]. The relevance of cPLA_2_α membrane bending in terms of its cellular function is explored in several recent reports, supporting a role for cPLA_2_α-mediated membrane vesiculation via its catalytic activity. Experiments based on RNAi and acyltransferase inhibitors established a direct relationship between cPLA_2_α and the production of lysophosphatidylcholine [14]. Others have linked the catalytic activity of cPLA_2_α to the generation of Golgi-derived tubules and vesicles, suggesting that there may be a dual role for this enzyme in the maintenance of Golgi structure and trafficking [15,16]. Multiple studies have established a relationship between cPLA_2_α-dependent Golgi structures and cholesterol-enriched membranes, in agreement with our findings [13,16].

Finally, cPLA_2_α is known for its non-redundant role in the production of prostaglandins and eicosanoids in murine models of arthritis, asthma, and cerebral ischemia [3,4]. The analysis of cPLA_2_α knockout mice revealed that the animals were infertile but otherwise showed no major defects. If cPLA_2_α has an important function in the maintenance of the Golgi structure and Golgi-derived endosomal trafficking, it is unclear how the cPLA_2_α knockout mice survive. Other cPLA_2_ isoforms may compensate for the absence of cPLA_2_α and maintain its homeostatic functions. Such redundancy in endocytic signaling was recently confirmed when cPLA_2_ε was implicated in clathrin-independent endosomal trafficking, which was dependent on its catalytic activity [54]. Although few reports have considered the importance of the C2 domain in membrane bending induced by cPLA_2_α and cPLA_2_ε, we have shown that membrane penetration by the cPLA_2_α C2 domain and its subsequent oligomerization play a key role in this process. Our preliminary data shows that the cPLA_2_δ C2 domain also bends membranes and senses curvature (Figure 4G,H). It is possible that the cPLA_2_ε C2 domain also bends membranes, and this should be investigated in the future. Taken together, these recent findings suggest that the cPLA_2_α isoforms contribute to redundant cPLA_2_α-dependent membrane remodeling capabilities and prevent the cPLA_2_α knockout animals from showing appreciable symptoms in the absence of cPLA_2_α.

## 5. Conclusions

This study provides new insight into the generation of membrane curvature by C2 domains based on the analysis of cPLA_2_α in vitro and in cells. We found that the cPLA_2_α C2 domain oligomerizes and senses membrane curvature, preferentially targeting vesicles with a high degree of positive curvature. Given that some of these vesicles are lipid droplets and some are comparable to those derived from the trans-Golgi network, this targeting is consistent with the cellular localization and in vivo function of cPLA_2_α. The selective targeting and oligomerization of cPLA_2_α on membranes of positive curvature is supported by studies showing that cPLA_2_α is involved in Golgi tubulation and vesiculation [13,15]. We provided evidence for a new class of membrane-bending enzymes, where lipid asymmetry and C2 domain penetration and oligomerization may work together or independently to maintain the membrane structure of the Golgi. Our proposed oligomerization mechanism should be tested for other C2 domains that induce membrane curvature alone despite the presence of tandem C2 domains in the protein.
